# Cytomegalovirus viraemia and mortality in renal transplant recipients in the era of antiviral prophylaxis. Lessons from the western Australian experience

**DOI:** 10.1186/s12879-017-2599-y

**Published:** 2017-07-17

**Authors:** Linda A. Selvey, Wai H. Lim, Peter Boan, Ramyasuda Swaminathan, Claudia Slimings, Amy E. Harrison, Aron Chakera

**Affiliations:** 1 School of Public Health, Curtin University, Bentley, WA Australia; 2 ANZDATA Registry, Adelaide, Australia; 3 Department of Renal Medicine, Sir Charles Gairdner Hospital, Perth, WA Australia; 4 Department of Infectious Diseases, Fiona Stanley Hospital, Murdoch, WA Australia; 5 Department of Microbiology, PathWest Laboratory Medicine, Perth, WA Australia; 6 Department of Nephrology and Renal Transplantation, Fiona Stanley Hospital, Murdoch, WA Australia; 7 North Ryde, NSW Australia; 8 Translational Renal Research Group, Harry Perkins Institute of Medical Research, QEII Medical Centre, Nedlands, WA Australia

**Keywords:** Cytomegalovirus reactivation, Renal transplantation, Mortality, Risk factors

## Abstract

**Background:**

Cytomegalovirus (CMV) establishes a lifelong infection that is efficiently controlled by the immune system; this infection can be reactivated in case of immunosuppression such as following solid organ transplantation. CMV viraemia has been associated with CMV disease, as well as increased mortality and allograft failure. Prophylactic antiviral medication is routinely given to renal transplant recipients, but reactivation during and following cessation of antiviral prophylaxis is known to occur. The aims of this study were to assess the incidence, timing and impact of CMV viraemia in renal transplant recipients and to determine the level of viraemia associated with adverse clinical outcomes.

**Methods:**

Data from all adult (18 years and over) Western Australian renal transplant recipients transplanted between 1 January 2007 and 31 December 2012 were obtained from the Australia and New Zealand Dialysis and Transplant registry and were supplemented with data obtained from clinical records. Potential risk factors for detectable CMV viraemia (≥600 copies/ml) and all-cause mortality were assessed using univariable analysis and Cox Proportional Hazards Regression.

**Results:**

There were 438 transplants performed on 435 recipients. The following factors increased the risk of CMV viraemia with viral loads ≥600 copies/ml: Donor positive/Recipient negative status; receiving a graft from a deceased donor; and receiving a graft from a donor aged 60 years and over. CMV viraemia with viral loads ≥656 copies/ml was a risk factor for death following renal transplantation, as was being aged 65 years and above at transplant, being Aboriginal and having vascular disease. Importantly 37% of the episodes of CMV viraemia with viral loads ≥656 copies/ml occurred while the patients were expected to be on CMV prophylaxis.

**Conclusions:**

CMV viraemia (≥656 copies/ml) was associated with all-cause mortality in multivariable analysis, and CMV viraemia at ≥656 copies/ml commonly occurred during the period when renal transplant recipients were expected to be on antiviral prophylaxis. A greater vigilance in monitoring CMV levels if antiviral prophylaxis is stopped prematurely or poor patient compliance is suspected could protect some renal transplant recipients from adverse outcomes such as premature mortality.

## Background

Cytomegalovirus (CMV) infection has long been recognised as a cause of morbidity and premature mortality amongst solid organ transplant recipients [1]. In Australia, the prevalence of CMV infection is estimated to be 57% among people up to 60 years of age [2]. The risks of CMV-related complications in kidney transplant recipients vary according to the serostatus of the donor (D) and recipient (R), with kidneys from CMV positive donors (D^+^) transplanted into CMV negative recipients (R^−^) carrying the highest risk [1]. Reactivation of CMV in kidney transplant recipients may manifest across a clinical spectrum from asymptomatic viraemia to tissue-invasive disease [3]. Epidemiological studies have also linked CMV to chronic allograft injury, accelerated atherosclerosis and malignancy, potentially caused by direct viral cytopathic effects as well as immune modulation induced by the virus [4–6].

CMV prophylaxis with antivirals, such as ganciclovir or valganciclovir, has been in use for a number of years in Australia and elsewhere. The current Australian guidelines recommend 3 months of antiviral prophylaxis for D^+^/R^+^ and D^−^/R^+^ recipients, 6 months for D^+^/R^−^ recipients, and none if both donor and recipient were negative for CMV [7]. CMV prophylaxis has been shown to reduce the risk of CMV disease and all-cause mortality in renal transplant recipients [8].

Despite the use of antiviral prophylaxis, CMV viraemia in transplant recipients remains a significant concern, with up to 35% of patients who were provided with prophylaxis becoming viraemic within 12 months of transplantation [1, 9]. In addition to the cost of prophylaxis and the risks of side-effects, in particular neutropenia, a concern is the development of late-onset disease (CMV viraemia occurring following cessation of prophylaxis), which is associated with allograft rejection [10] and increased mortality [11, 12].

Although a number of laboratory techniques exist for the detection of CMV viraemia, most centres across Australia are now performing quantitative nucleic acid testing (NAT). While very high levels of viraemia are typically associated with the presence of CMV syndrome or tissue-invasive disease, the clinical significance of lower-level viraemia remains unclear [13], and differences in NAT assays can lead to significant discrepancies in the quantification of viraemia between laboratories [14].

The aims of this study were to assess the incidence, timing and impact of CMV viraemia in renal transplant recipients and to determine the level of viraemia associated with adverse clinical outcomes.

## Methods

### Study population

All adult (age ≥ 18 years) patients who received a live or deceased donor kidney transplant in Perth, Western Australia (WA), between January 2007 and December 2012 were included in this study. Data were extracted from local health records and linked to data recorded in the Australia and New Zealand Dialysis and Transplant (ANZDATA) registry. If a patient received more than one kidney transplant during the study period, data regarding both grafts were extracted and both transplants were considered as separate grafts for analysis.

In WA, most patients received basiliximab induction and routine triple immunosuppression with a calcineurin inhibitor (CyA/tacrolimus), mycophenolate sodium or mycophenolate mofetil and oral prednisolone. Patients at lower risk of rejection did not receive either basiliximab or thymoglobulin induction and those at highest risk (around 10%) received thymoglobulin induction instead of basiliximab. Target tacrolimus trough levels are typically between 5 and 10 μg/L for the first 3 months, with the dose of prednisolone reduced to 5–10 mg daily by 3 months. Biopsy-proven T cell mediated rejection episodes were managed with intravenous methylprednisolone, with T cell depleting antibody being prescribed for steroid-resistant rejection episodes.

CMV prophylaxis with valganciclovir was administered for 100 days to R^+^ patients, 200 days to D^+^/R^−^ grafts, and no prophylaxis was given to D^−^/R^−^ grafts. CMV testing at both centres during or after prophylaxis is usually performed on a monthly basis throughout the first year post-transplant. After that time, testing was done on an adhoc basis, usually only if there was a clinical indication. Valganciclovir at induction doses was routinely given following CMV reactivation at a viral load between 10^3^ and 10^5^ copies/mL. CMV treatment was routinely stopped after CMV was undetectable for two consecutive tests over at least a 1 month period.

### Data collection

The following data were obtained from ANZDATA (to December 2013): age at transplantation; graft number; date of transplantation; date of birth; racial origin; weight and height at transplant; maximum panel reactive antibody (PRA) status; recipient smoking status; recipient CMV serology status; donor source; donor age; human leukocyte antigen (HLA) mismatches; occurrence of acute rejection (including rejection type, severity and treatment); induction therapy (interleukin-2 receptor antibody or T cell depleting antibody); immunosuppression prescribed at 0, 3, 6 months and at each year post-transplant; comorbidities of cardiovascular disease, chronic lung disease, diabetes or cancer (post-transplant or recurrent); whether or not graft failure occurred and the date of failure; and whether or not the patient died (to December 2014) and the date of death. Information on treatment for CMV viraemia was not available.

Donor CMV serology status was obtained from individual patient charts at the two transplant hospitals. Recipient CMV serology status was confirmed from individual patient records and where differences between patient records and ANZDATA were identified, the patient record information was used. Any data missing from the ANZDATA dataset were also obtained from patient records where possible.

CMV DNA plasma viral load testing was performed at PathWest Laboratory Medicine WA using the COBAS Amplicor CMV MONITOR assay (Roche Diagnostics, Branchburg, N.J., USA) from January 2007 to 12 May 2011, which has a linear range of 6 × 10^2^ to 1 × 10^5^ copies/mL (not referenced to the international CMV quantitative standard, so not reportable in IU/mL). From 13 May 2011 the Abbott RealT*ime* CMV assay (Abbott Molecular Inc., Des Plaines, IL, USA) on the m2000 RealT*ime* platform was introduced, which has a linear range of 2 × 10^1^ to 1 × 10^8^ copies/mL (multiplication of copies/mL by 1.56 converts the result to IU/mL according to the WHO international CMV quantitative standard). We obtained testing data (date of test, test result) up to December 2014 inclusive and CMV results were reported in copies/ml.

### Data analysis

Incidence rates and 95% confidence intervals were calculated for all variables of interest for the outcomes: CMV viraemia with greater than or equal to 600 copies/ml; and patient death prior to 31 December 2014. The start time was the date of the first recorded transplant for calculating the incidence of CMV viraemia; and the date of the most recent recorded transplant for calculating the incidence of patient death prior to 2014. When calculating incidence for time-dependent variables, the start time was the time of transplant for the period when the variable equalled zero. If and when the categorical variables became greater than zero, this was the start time for incidence. Patient days prior to that time were counted as patient days for that variable being zero. The Exact method was used to estimate *p*-values for the significance of effect for binomial variables and univariable Cox Proportional Hazards analysis was used to estimate *p*-values for categorical variables with more than two possible values. Time-dependent variables, such as CMV, were fitted as time-dependent variables in the univariable Cox Proportional Hazards models. Age at transplantation was categorised to <65 and ≥65 on the basis of 65 being an age cut-off for increased risk of death and adverse outcomes in other studies [15–17]. The cut-off for categorisation of CMV levels was set at 600 copies/ml on the basis that 600 copies/ml was the limit of detection for the less sensitive Roche COBAS Amplicor assay. The cut-off for categorisation of donor age was set at 60 years of age, and for categorisation of the total number of HLA mismatches the cut-off was set at two. Patient death outcome data were censored as of December 2014 and CMV viraemia outcome data were censored as of December 2013. The earlier censoring of CMV viraemia outcome data was because information about rejection and immunosuppression was only available up to December 2013.

Multivariable survival analysis was performed using Cox Proportional Hazards regression. Tied failures were calculated using the Breslow method for ties. Robust standard errors were used to estimate *p* values and 95% confidence intervals. Covariates with a *p*-value of 0.100 or less in the univariable models or covariates previously reported to correlate with study outcomes were included in the initial models. Variables were excluded from the model if their *p*-values for their adjusted hazard ratio was >0.05. For the model analysing CMV viraemia as the outcome, immunosuppression was categorised into three groups: calcineurin inhibitors (cyclosporin A or tacrolimus); antimetabolites (azathioprine, mycophenolate mofetil, or mycophenolate sodium); and mammalian target of rapamycin (mTOR) inhibitors (everolimus or sirolimus), and fitted as time-dependent covariates for each time period for which immunosuppression data were recorded in ANZDATA (3 months, 6 months, 1, 2, 3, 4, and 5 years). Anti-T lymphocyte antibody treatment after transplant was fitted as a time-dependant variable and was compared to having no treatment or treatment at or before transplantation. All rejection episodes were biopsy-proven with severe rejection defined as any vascular rejection or any severe humoral, glomerular or cellular rejection. Rejection variables were also fitted as time-dependent covariates. The following variables were included in the initial model for CMV viraemia: recipient age (categorised at 65 years), sex, donor age (categorised at 60 years), donor/recipient CMV status, immunosuppression, rejection, severe rejection, numbers of HLA mismatches (categorised as two or less or greater than two), and donor status (deceased or live). For the model examining death as the outcome, CMV status was fitted as a time-dependent covariate as one or more episodes (combined). The variables included in the initial model for death as an outcome were age (categorised at 65 years), sex, donor age (categorised at 60 years), donor status (deceased or live), rejection, severe rejection, CMV status, Aboriginality, smoking status, graft failure and the following comorbidities: diabetes; cerebrovascular disease, any cardiovascular disease other than cerebrovascular disease in patients with no cerebrovascular disease; chronic lung disease and cancer. The proportional hazards assumption was tested for all variables in every final model based on the analysis of Schoenfeld residuals.

A Kaplan-Meier plot showing death by CMV viral load was generated with CMV viral load being fitted as a time-dependent variable.

Data analysis was performed using STATA v14 (StataCorp LP, College Station, Texas, USA).

## Results

There were 438 renal transplants performed in 435 recipients (with three patients receiving a second transplant in the study period) in WA from January 2007 to December 2012 and all cases were included in the analysis. The median age at transplant was 51 years (range 18–80).

### Risk factors associated with CMV viraemia

**Fig. 1:**
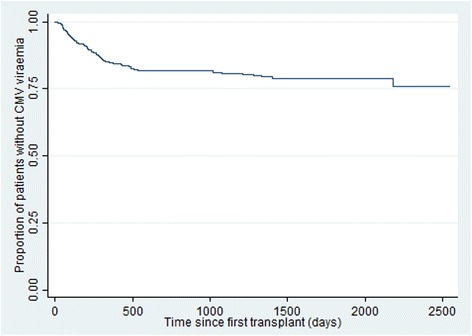
Kaplan Meir survival plot for onset of CMV viraemia ≥600 copies/ml after first transplantation

One hundred and thirty four (30.6%) patients had at least one episode of detectable viraemia during the study period. Of these, 74 (55.2%) patients had at least one episode of viraemia at 600 copies/ml or more. Eleven (14.9%) of the 74 patients had two episodes of CMV viraemia at 600 copies/ml or more with intervening periods of no CMV DNA being detected. One patient had a single CMV viraemia episode of 600 copies/ml or more before transplantation. This viraemic episode was excluded, leaving in the analysis 84 CMV viraemia episodes of 600 copies/ml or more from 73 patients over 461,961 patient days. The incidence of CMV viraemia of 600 copies/ml or more was 18.2 per 100,000 person days (95%CI 14.7–22.5). Forty-six patients died before 31 December 2013 (Fig. 1). There were three patients who developed CMV viraemia after graft failure and had no record of a subsequent transplant. These patients were excluded from the univariable analysis, and the CMV episodes were excluded from the Cox Proportional Hazards analysis as they were censored at graft failure.

Table 1 presents the numbers and rates of CMV viraemia at 600 copies/ml or more stratified by covariate categories. Being 65 years or above at the time of transplant, having D^+^/R^−^ status, having at least one episode of severe rejection, and having a transplant from a deceased donor or a donor aged 60 years and over were all significantly associated with having CMV viraemia in univariable analysis (Table 1).

**Table 1:** CMV proportions and rates by covariates

	Total n	Number with CMV (%)	CMV rate per 100,000 patient days (95% CI)	*p*-value
Total	435	70 (16.1)	18.2 (14.7, 22.5)	
Sex				
Female	164	27 (16.5)	19.1 (13.6, 26.7)	0.893
Male	272	43 (15.9)	17.6 (13.4, 23.3)	
Age 65 and over				
< 65	396	58 (14.7)	16.4 (13.0, 20.7)	0.019
≥ 65	39	12 (30.8)	46.1 (26.8, 79.4)	
Transplant Hospital				
SCGH	232	33 (14.2)	14.60 (10.58, 20.16)	0.296
RPH	206	40 (19.4)	22.53 (16.93, 29.99)	
Index graft history				
First graft	384	64 (16.7)	18.8 (15.0, 23.5)	0.425
Had previous graft(s)	51	6 (11.8)	14.1 (7.0, 28.2)	
Number of grafts during follow up				
Single graft	432	69 (16.0)	18.0 (14.5, 22.3)	0.410
Two grafts	3	1 (33.3)	191.2 (26.9, 1357.4)	
Donor & recipient CMV status (*n* = 430)				
D^−^R^−^	49	0 (0)	0	<0.001
D^−^R^+^	115	13 (11.3)	11.0 (6.5, 18.6)	
D^+^R^−^	68	25 (36.8)	56.8 (40.0, 80.8)	
D^+^R^+^	195	32 (16.4)	18.9 (13.8, 25.9)	
HLA mismatch (index graft) (*n* = 432)				
< 2 mismatches	58	5 (8.6)	8.3 (3.7, 18.5)	0.153
≥ 2 mismatches	371	65 (17.5)	20.2 (16.2, 25.2)	
Antibody treatment				
None or before or at transplant only	394	59 (30.3)	15.0 (8.5, 26.5)	
Post-transplant	33	11 (33.3)	18.8 (15.0, 23.7)	0.687
Rejection (any)				
No	342	51 (14.9)	16.3 (12.6, 21.1)	0.205
Yes	93	19 (20.4)	35.3 (16.6, 35.3)	
Rejection (severe)				
No	407	63 (15.5)	16.7 (13.3, 21.1)	0.002
Yes	28	7 (25.0)	38.3 (21.7, 67.4)	
Aboriginal				
No	386	63 (16.3)	18.3 (14.6, 22.9)	0.838
Yes	49	7 (14.3)	17.6 (9.2, 33.8)	
Donor aged >60				
No	317	40 (12.6)	12.9 (9.6, 17.2)	0.002
Yes	118	30 (25.2)	36.4 (26.5, 50.0)	
Deceased donor				
No	192	25 (13.0)	11.4 (7.8, 16.8)	0.017
Yes	244	45 (18.5)	24.8 (19.2, 32.1)	

#### Multivariable analysis

For the Cox Proportional Hazards regression analysis, the three CMV episodes after transplant failure were excluded as the data were censored at transplant failure. CMV-negative recipients receiving grafts from CMV-negative donors were also excluded from the analysis, as none of these participants developed CMV viraemia. All three patients with a second graft during the follow up period exited the survival model at the failure date of the first graft. Having D^+^/R^−^ CMV status was associated with a five-fold increased risk of CMV viraemia of 600 copies/ml or more. Receiving a graft from a donor aged 60 or more, and having a graft from a deceased donor, were also significantly associated with CMV viraemia of 600 copies/ml or more. Immunosuppressive regimens that included an mTOR inhibitor (either everolimus or sirolimus) resulted in a 70% decreased risk of a CMV viraemic episode (as a time-dependent covariate), although this was not statistically significant (*p* = 0.052) (Table 2).

**Table 2:** Final Cox Proportional Hazards regression model for CMV incidence (with mTOR inhibitor fitted as a time dependent variable)

Variable	Adjusted Hazard Ratio (95% CI)	*p*-value
Recipient aged ≥65 at transplant	1.85 (0.96, 3.58)	0.067
Donor aged ≥60	2.24 (1.36, 3.67)	0.001
Deceased donor	2.03 (1.18, 3.50)	0.011
Donor/Recipient CMV status		
D^−^/R^+^ (reference)	−	−
D^+^/R^−^	5.44 (2.49, 11.89)	<0.001
D^+^/R^+^	1.73 (0.84, 3.56)	0.138
mTOR inhibitor	0.31 (0.09, 1.02)	0.054

### Association between CMV viraemia and all-cause mortality

Of the 438 transplant recipients, 55 (12.6%) died before 31 December 2014. Having at least one episode of CMV detectable at 600 copies/ml or greater, being aged 65 or over at the time of transplant, receiving a graft from a deceased donor, lung disease, cerebrovascular disease, and Aboriginal race were all predictors of death in univariable analysis (Table 3).

**Table 3:** Univariable analysis of potential predictors of death amongst transplant recipients (*n* = 438)

Variable	Number	Number of deaths (%)	Death rate per 100,000 patient days (95% CI)	*p*-value
All cases	438	55 (12.6)	7.9 (6.0, 10.2)	
Transplant Hospital				
SCGH	232	29 (12.5)	7.9 (5.5, 11.3)	1.0
RPH	206	26 (12.6)	7.8 (5.3, 11.5)	
CMV viral load of ≥600 copies/ml				
No	364	35 (9.6)	5.8 (4.2, 8.0)	<0.001
Yes	74	20 (27.0)	21.0 (13.6, 32.6)	
CMV viral load (copies/ml)				
< 600	364	35 (9.6)	5.8 (4.2, 8.0)	
600–4999	42	10 (23.8)	18.7 (9.7, 35.9)	<0.001
≥ 5000	32	10 (31.3)	23.5 (13.0, 42.4)	0.001
Sex				
Female	166	25 (15.1)	9.2 (6.2, 13.7)	0.30
Male	272	30 (11.0)	7.0 (4.9, 10.0)	
Deceased donor				
No	192	17 (8.9)	5.2 (3.2, 8.3)	0.02
Yes	246	38 (15.5)	10.2 (7.4, 14.0)	
Donor aged ≥60				
No	317	35 (11.0)	6.8 (4.9, 9.5)	0.10
Yes	121	20 (16.5)	10.8 (7.0, 16.8)	
Graft failure				
No	390	47 (12.1)	7.6 (5.7, 10.1)	0.51
Yes	48	8 (16.7)	9.7 (4.9, 19.4)	
Age ≥ 65 at transplant				
No	399	42 (10.5)	6.5 (4.8, 8.8)	<0.001
Yes	39	13 (33.3)	24.2 (14.1, 41.7)	
Lung Disease				
No	378	41 (10.9)	6.8 (5.0, 9.2)	0.02
Yes	60	14 (23.3)	14.6 (8.6, 24.6)	
Cerebrovascular disease				
No	407	41 (10.1)	6.2 (4.6, 8.4)	<0.001
Yes	31	14 (45.1)	34.5 (20.4, 58.3)	<0.001
Vascular disease other than cerebrovascular				
No	104	36 (10.8)	6.7 (4.8, 9.3)	0.05
Yes	334	19 (18.3)	11.7 (7.5, 18.4)	
Diabetes				
No	293	35 (12.0)	7.5 (5.4, 10.4)	0.61
Yes	145	20 (13.8)	8.6 (5.6, 13.3)	
HLA mismatch ≥2				
No	373	6 (10.2)	5.9 (2.6, 13.1)	0.45
Yes	59	49 (13.1)	8.3 (6.2, 10.9)	
Maximum Panel Reactive Antibody level ≥ 50%				
No	366	46 (12.6)	7.8 (5.8, 10.4)	0.62
Yes	61	9 (14.8)	9.2 (4.8, 17.7)	
Any cancer diagnosis				
No	352	42 (11.9)	7.5 (5.5, 10.2)	0.51
Yes	86	13 (15.1)	9.2 (5.3, 15.8)	
Aboriginal				
No	387	40 (10.3)	6.4 (4.7, 8.8)	0.001
Yes	51	15 (29.4)	19.3 (11.6, 32.0)	
Any rejection				
No	302	37 (12.3)	7.9 (5.7, 10.9)	0.99
Yes	136	18 (13.2)	7.8 (4.9, 12.4)	
Severe rejection				
No	404	49 (12.1)	7.7 (5.8, 10.1)	0.54
Yes	34	6 (17.7)	9.9 (4.4, 21.9)	
Healthy weight (BMI 19–25) at transplant				
No	272	35 (12.9)	8.3 (5.9, 11.5)	0.78
Yes	159	20 (12.6)	7.6 (4.9, 11.8)	
Current smoker				
No	398	48 (12.1)	7.6 (5.7, 10.1)	0.45
Yes	40	7 (17.5)	10.2 (4.9, 21.5)	

### Sensitivity analysis for CMV viraemia and mortality

**Fig. 2:**
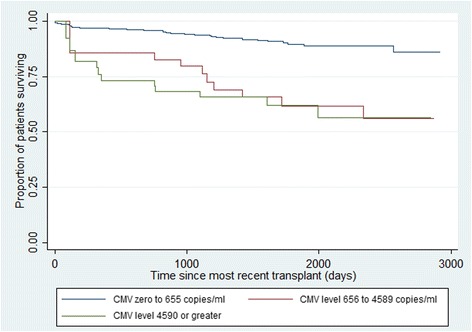
Kaplan Meir survival plots for all-cause deaths according to peak CMV viral load after most recent transplantation

We next sought to examine the relationship between a number of thresholds for CMV viral load, the duration of viraemia and all-cause mortality. There were 134 (30.6%) patients who had at least one episode of detectable CMV viraemia. The median CMV viral load among these cases was 656 copies/ml (IQR 68, 4590), and levels ranged from 2.6 to 5,710,000 copies/ml. Using the quartile values as cut-offs, the CMV viral load was categorised as <68, 68–655, 656–4589 and 4590+ copies/ml. A Cox Regression model showed a significant HR for death only for those cases with a CMV viral load of ≥656 copies/ml (Table 4). Kaplan Meier survival plots for death by CMV viral load are shown in Fig. 2.

**Table 4:** Cox Proportional Hazards Regression analysis for death by CMV level

Variable	Adjusted Hazard Ratio (95% CI)	*p*-value
CMV viral load <68 copies/ml (reference)	-	-
CMV viral load 68–655 copies/ml	0.63 (0.15, 2.62)	0.52
CMV viral load 656–4589 copies/ml	2.35 (1.12, 4.91)	<0.001
CMV viral load ≥4590 copies/ml	3.19 (1.61, 6.31)	0.001

### Multivariable analysis for factors associated with all cause mortality

Using Cox Proportional Hazards regression analysis, having at least one episode of CMV viraemia with viral loads of 656 copies/ml or more, Aboriginal race, aged 65 or more at transplant, having cerebrovascular disease, and having any other vascular disease were all associated with death (Table 5). An interaction term of CMV with age was not significant and were therefore not included in the final model.

**Table 5:** Final Cox Proportional Hazards regression model for death

Variable	Adjusted Hazard Ratio (95% CI)	*p*-value
CMV viral load		
Less than 656 copies/ml (reference)	-	
656–4590 copies/ml	3.25 (1.42, 7.44)	0.007
≥ 4590 copies/ml	3.94 (1.75, 8.89)	0.012
Aboriginal	3.39 (1.74, 6.63)	<0.001
Age ≥ 65 at transplant	2.39 (1.10, 5.18)	0.027
Cerebrovascular disease	4.21 (1.81, 9.78)	0.001
Other vascular disease	2.21 (1.19, 4.09)	0.012

### Multivariable analysis for factors associated with death with a functioning graft

Of the 55 patients who died, 47 died with a functioning graft. A new Cox Proportional Hazards regression model was generated censoring at graft failure the eight cases who died after graft failure. In this model, having at least one episode of CMV viraemia with levels of 656–4589 copies/ml (aHR 3.36, 95% CI 1.49, 7.55, *p* = 0.003), having at least one episode of CMV viraemia with levels of 4590 copies/ml or more (aHR 3.96, 95% CI 1.85, 8.49, *p* < 0.001), Aboriginal race (aHR 3.33, 95% CI 1.74, 6.36, *p* < 0.001), aged ≥65 at transplant (aHR 2.38, 95% CI 1.11, 5.13, *p* = 0.026), cerebrovascular disease (aHR 4.24, 95% CI 1.87, 9.66, *p* = 0.001), and other vascular disease (aHR 2.23, 95% CI 1.22, 4.08, *p* = 0.009) were associated with death with a functioning graft.

### CMV duration

The duration of CMV viraemia was not associated with death. The incidence of death from most recent transplant was 5.8 (95% CI 4.2, 8.1) for patients with no episode of CMV viraemia of 656 copies/ml or more; 20.9 (95% CI 11.2, 38.8) for patients with CMV viraemia of 656 copies/ml or more for a duration of up to 7 days; and 21.0 (95% CI 11.3, 39.1) for patients with CMV viraemia of 656 copies/ml or more for a duration of at least 7 days. Further analysis comparing death with duration of CMV viraemia with a threshold of 14 days and with 28 days also did not show an association between duration of CMV viraemia and death (data not shown).

### Onset of CMV viraemia

There were 73 patients who had at least one episode of CMV viraemia of 656 copies/ml or greater post-transplant. Eleven of these patients had two episodes of CMV viraemia of 656 copies/ml or greater (a total of 84 episodes in 73 patients). Of those viraemic episodes, 31 (37%) occurred during the period when the patient was expected to be on CMV prophylaxis.

## Discussion

In this contemporary group of kidney transplant recipients, CMV viraemia with a threshold of at least 656 copies/ml was associated with over a three-fold greater risk of all-cause mortality and death with functioning graft. An important observation is that 37% of CMV viraemia of at least 656 copies/ml occurred during the period of presumed antiviral prophylaxis suggesting that close monitoring of CMV viraemia is warranted should patients cease antiviral prophylaxis prematurely or if non-compliance is suspected. Our finding that CMV viraemia of relatively low levels (≥656 copies/ml) was also associated with death also suggests vigilance with CMV treatment when CMV viraemia reaches these levels. Although we had expected that increasing duration of CMV viraemia would be associated with mortality, this was not apparent in this study. However, the inconsistent timing of CMV testing may have modified the association between duration of CMV viraemia and outcome. Our study builds on the work of previous investigators [11, 12, 18–22], by being conducted at a time when all eligible renal transplant recipients were prescribed CMV prophylaxis, and by considering risk factors and consequences of CMV viraemia, rather than CMV disease.

Given that having CMV activation at viral loads of 656 copies/ml or greater is associated with death, our finding that 37% of the episodes of CMV activation at this level occurred while the patient was expected to be on CMV prophylaxis is of great concern. We were unable to determine whether this was due to a lack of compliance with CMV prophylaxis, or whether prophylaxis was withdrawn due to intolerable side effects. Our findings do highlight the critical importance of monitoring CMV viral loads during periods when antiviral prophylaxis has been prematurely ceased, and the provision of fairly aggressive CMV treatment should levels rise over 600 copies/ml. They also stress the importance of patient compliance with CMV prophylaxis and of ensuring dose adjustment of prophylaxis according to the patient’s glomerular filtration rate.

While the latest CMV assays are extremely sensitive and are able to detect low-level viraemia, it is unknown whether low-level viraemia below the threshold of earlier assays is clinically significant. Only levels of at least 656 copies/ml were associated with mortality in our analysis. While other studies have shown an association between acute rejection and risk of CMV viraemia, it was unclear whether the association between rejection and CMV viraemia was related to the increased dose of immunosuppression or a direct effect of the rejection process [22].

The association between Aboriginal status and death following transplantation has been previously described. Due to a range of factors, Aboriginal people are more likely to develop chronic renal failure and they have poorer outcomes than non-Aboriginal Australians post-transplant [23].

The association between the use of mTOR inhibitors and reduced rates of viral infections has been shown in several studies, including a recent meta-analysis in kidney transplant recipients [24, 25]. Although the use of mTOR inhibitors was associated with a reduced risk of CMV viraemia in the unadjusted model, this association was no longer present in the adjusted model, which may suggest a type II error or that reduced intensity of immunosuppression (as a result of eliminating calcineurin inhibitors) may be a more important factor in reducing the risk of CMV viraemia.

Receiving a graft from a donor aged 60 years and older has previously been found to be associated with late-onset CMV disease in D^+^/R^−^ patients [18]. To our knowledge, there are no other studies finding an association between receiving a graft from a deceased donor and CMV viraemia/disease. An interaction term between the variables, donor aged ≥60 years and deceased donor, was not significant in the model. While we did not find that the number of HLA mismatches between donor and recipient was associated with CMV viraemia, our finding may also be related to a reduced level of immunological matching between recipients and deceased donors compared to between recipients and live donors, therefore increasing the level of immunosuppression required to prevent rejection.

It is known that a high and rapid increase of plasma CMV viral load is associated with a greater likelihood of invasive CMV disease [13], however, CMV disease can occur even with only low levels of viral DNA detectable in plasma, as described with gastrointestinal and retinal disease [26]. There is no recognised cut-off for the commencement of antiviral therapy; many earlier studies were hampered by use of assays with no reference to the WHO international quantitative standard [13]. One recent study of CMV seropositive kidney, heart and liver transplant recipients found a high predictive value for CMV disease in those with CMV plasma viral load of 3983 IU/mL or more compared with lower levels [27]. Lower CMV viral loads not associated with a risk of invasive CMV disease may still be associated with the indirect effects of CMV. However, with a poor understanding of how antiviral therapy alters the indirect effects of CMV and the association of CMV viral loads with indirect CMV effects, there are no definite recommendations regarding antiviral therapy for this indication [28, 29].

There are several strengths and limitations to our study. This is one of the largest studies to evaluate the association between CMV viraemia and clinical outcomes in the era where antiviral prophylaxis is standard practice. Even though this is not a small study, our study may have had insufficient power to examine the association between CMV viraemia and several outcomes including death due to different causes. ANZDATA and patient records do not document the duration and dose of CMV prophylaxis and there is limited recording of patient compliance. Another limitation of our study is that the results in copies/mL for different CMV viral load assays used in this study are not directly comparable without being able to report IU/mL for both assays [13]. For the COBAS Amplicor assay the only available data relating copies/mL to IU/mL by the CMV international quantitative standard is provided by indirect comparison to the COBAS Ampliprep assay [30].

## Conclusions

We found that CMV viraemia (at 656 copies/ml or greater) was associated with over a three-fold increased risk of all-cause mortality amongst renal transplant recipients. An association between CMV disease and all-cause mortality has been previously shown, but less frequently for CMV viraemia alone [1, 11, 18, 20, 22, 31, 32], although these findings are not consistent across all studies [12]. Despite the routine use of antiviral prophylaxis after kidney transplantation in R+ and D+/R- recipients, 37% of recipients experienced CMV viraemia during the recommended period for antiviral prophylaxis. Clinicians should be cognisant of the need to monitor and identify CMV viraemia when prophylaxis is prematurely ceased or non-compliance is suspected. Further examination of the association between duration of CMV viraemia and patient outcomes in larger cohorts is required. While some cases of CMV disease were recorded in the ANZDATA, there is not a specific data field where this can be documented and it is likely that this was significantly under-reported. Therefore, we have not assessed the relationship between CMV disease and transplant outcomes or factors associated with CMV disease. We were unable to determine whether or not our findings reflect a true impact of CMV viraemia on mortality, or whether CMV viraemia is a confounder for other, unmeasured factors associated with death [32].

## Abbreviations

aHR: adjusted hazard ratio
ANZDATA: Australia and New Zealand Dialysis and Transplant registry
CI: Confidence interval
CMV: Cytomegalovirus
D^−^: Donor has negative CMV serology
D^+^: Donor has positive CMV serology
HLA: Human leukocyte antigen
HR: Hazard ratio
IQR: Interquartile range
NAT: Quantitative nucleic acid testing
PRA: Panel reactive antibody
R^−^: Recipient has negative CMV serology prior to transplantation
R^+^: Recipient has positive CMV serology prior to transplantation
WA: Western Australia

## References

[1] Fisher RA (2009). Cytomegalovirus infection and disease in the new era of immunosuppression following solid organ transplantation. Transpl Infect Dis.

[2] Seale H, MacIntyre CR, Gidding HF, Backhouse JL, Dwyer DE, Gilbert L (2006). National serosurvey of cytomegalovirus in Australia. Clin Vaccine Immunol.

[3] Patel R, Paya CV (1997). Infections in solid-organ transplant recipients. Clin Microbiol Rev.

[4] Freeman RB (2009). The 'indirect' effects of cytomegalovirus infection. Am J Transplant.

[5] Fishman JA (2013). Overview: cytomegalovirus and the herpesviruses in transplantation. Am J Transplant.

[6] Courivaud C, Bamoulid J, Chalopin JM, Gaiffe E, Tiberghien P, Saas P, Ducloux D (2013). Cytomegalovirus exposure and cardiovascular disease in kidney transplant recipients. J Infect Dis.

[7] Pilmore H, Pussell B, Goodman D (2011). KHA-CARI guideline: cytomegalovirus disease and kidney transplantation. Nephrology.

[8] Hodson EM, Ladhani M, Webster AC, Strippoli GF, Craig JC. Antiviral medications for preventing cytomegalovirus disease in solid organ transplant recipients. *Cochrane Database Syst Rev*. 2013:CD003774. doi:10.1002/14651858.CD003774.pub4. 10.1002/14651858.CD003774.pub4 23450543

[9] Humar A, Lebranchu Y, Vincenti F, Blumberg EA, Punch JD, Limaye AP, Abramowicz D, Jardine AG, Voulgari AT, Ives J (2010). The efficacy and safety of 200 days valganciclovir cytomegalovirus prophylaxis in high-risk kidney transplant recipients. Am J Transplant.

[10] Razonable RR, Rivero A, Rodriguez A, Wilson J, Daniels J, Jenkins G, Larson T, Hellinger WC, Spivey JR, Paya CV (2001). Allograft rejection predicts the occurrence of late-onset cytomegalovirus (CMV) disease among CMV-mismatched solid organ transplant patients receiving prophylaxis with oral ganciclovir. J Infect Dis.

[11] Harvala H, Stewart C, Muller K, Burns S, Marson L, MacGilchrist A, Johannessen I (2013). High risk of cytomegalovirus infection following solid organ transplantation despite prophylactic therapy. J Med Virol.

[12] San-Juan R, De Dios B, Garcia-Reyne A, Fernandez-Ruiz M, Lumbreras C, Lopez-Medrano F, Morales JM, Hernando S, Folgueira D, Jimenez C, Aguado JM (2013). Limited impact of cytomegalovirus infection in the long-term outcome of renal and liver transplant. J Clin Virol.

[13] Razonable RR, Hayden RT (2013). Clinical utility of viral load in management of cytomegalovirus infection after solid organ transplantation. Clin Microbiol Rev.

[14] Pang XL, Fox JD, Fenton JM, Miller GG, Caliendo AM, Preiksaitis JK (2009). American Society of Transplantation infectious diseases Community of P, Canadian Society of T: Interlaboratory comparison of cytomegalovirus viral load assays. Am J Transplant.

[15] Veroux M, Grosso G, Corona D, Mistretta A, Giaquinta A, Giuffrida G, Sinagra N, Veroux P (2012). Age is an important predictor of kidney transplantation outcome. Nephrol Dial Transplant.

[16] Meier-Kriesche HU, Ojo AO, Cibrik DM, Hanson JA, Leichtman AB, Magee JC, Port FK, Kaplan B (2000). Relationship of recipient age and development of chronic allograft failure. Transplantation.

[17] Frei U, Noeldeke J, Machold-Fabrizii V, Arbogast H, Margreiter R, Fricke L, Voiculescu A, Kliem V, Ebel H, Albert U (2008). Prospective age-matching in elderly kidney transplant recipients--a 5-year analysis of the Eurotransplant senior program. Am J Transplant.

[18] Luan FL, Kommareddi M, Ojo AO (2011). Impact of cytomegalovirus disease in D+/R- kidney transplant patients receiving 6 months low-dose valganciclovir prophylaxis. Am J Transplant.

[19] Opelz G, Dohler B, Ruhenstroth A (2004). Cytomegalovirus prophylaxis and graft outcome in solid organ transplantation: a collaborative transplant study report. Am J Transplant.

[20] Desai R, Collett D, Watson CJ, Johnson PJ, Moss P, Neuberger J (2015). Impact of cytomegalovirus on long-term mortality and cancer risk after organ transplantation. Transplantation.

[21] Boudreault AA, Xie H, Rakita RM, Scott JD, Davis CL, Boeckh M, Limaye AP (2011). Risk factors for late-onset cytomegalovirus disease in donor seropositive/recipient seronegative kidney transplant recipients who receive antiviral prophylaxis. Transpl Infect Dis.

[22] Browne BJ, Young JA, Dunn TB, Matas AJ (2010). The impact of cytomegalovirus infection >/=1 year after primary renal transplantation. Clin Transpl.

[23] Rogers NM, Lawton PD, Jose MD (2006). Kidney transplant outcomes in the indigenous population in the northern territory of Australia. Transplantation.

[24] Lim WH, Eris J, Kanellis J, Pussell B, Wiid Z, Witcombe D, Russ GR (2014). A systematic review of conversion from calcineurin inhibitor to mammalian target of rapamycin inhibitors for maintenance immunosuppression in kidney transplant recipients. Am J Transplant.

[25] Brennan DC, Legendre C, Patel D, Mange K, Wiland A, McCague K, Shihab FS (2011). Cytomegalovirus incidence between Everolimus versus Mycophenolate in *de novo* renal transplants: pooled analysis of three clinical trials. Am J Transplant.

[26] Cummins NW, Deziel PJ, Abraham RS, Razonable RR (2009). Deficiency of cytomegalovirus (CMV)-specific CD8+ T cells in patients presenting with late-onset CMV disease several years after transplantation. Transpl Infect Dis.

[27] Martin-Gandul C, Perez-Romero P, Sanchez M, Bernal G, Suarez G, Sobrino M, Merino L, Cisneros JM, Cordero E (2013). Spanish network for research in infectious D: determination, validation and standardization of a CMV DNA cut-off value in plasma for preemptive treatment of CMV infection in solid organ transplant recipients at lower risk for CMV infection. J Clin Virol.

[28] Razonable RR, Humar A (2013). Cytomegalovirus in solid organ transplantation. Am J Transplant.

[29] Torre-Cisneros J, Aguado JM, Caston JJ, Almenar L, Alonso A, Cantisan S, Carratala J, Cervera C, Cordero E, Farinas MC (2016). Management of cytomegalovirus infection in solid organ transplant recipients: SET/GESITRA-SEIMC/REIPI recommendations. Transplant Rev (Orlando).

[30] Pritt BS, Germer JJ, Gomez-Urena E, Bishop CJ, Mandrekar JN, Irish CL, Yao JD (2013). Conversion to the COBAS AmpliPrep/COBAS TaqMan CMV test for management of CMV disease in transplant recipients. Diagn Microbiol Infect Dis.

[31] Arthurs SK, Eid AJ, Pedersen RA, Kremers WK, Cosio FG, Patel R, Razonable RR (2008). Delayed-onset primary cytomegalovirus disease and the risk of allograft failure and mortality after kidney transplantation. Clin Infect Dis.

[32] Santos CA, Brennan DC, Fraser VJ, Olsen MA (2014). Delayed-onset cytomegalovirus disease coded during hospital readmission after kidney transplantation. Transplantation.

